# Chlorogenic Acid Supplementation Benefits Zebrafish Embryos Exposed to Auranofin

**DOI:** 10.3390/pharmaceutics12121199

**Published:** 2020-12-11

**Authors:** Jasper Z. S. Chiu, Isabella Hold, Trent A. C. Newman, Julia A. Horsfield, Arlene McDowell

**Affiliations:** 1 School of Pharmacy, University of Otago, Dunedin 9016, New Zealand; czesiong@gmail.com (J.Z.S.C.); isabella.hold@gmail.com (I.H.); 2 Department of Pharmaceutical Technology, Institute of Pharmaceutical Sciences, Karl-Franzens University, Graz 8010, Austria; 3 Department of Pathology, University of Otago, Dunedin 9016, New Zealand; tacnewman@gmail.com (T.A.C.N.); julia.horsfield@otago.ac.nz (J.A.H.)

**Keywords:** *Sonchus oleraceus*, chlorogenic acid, zebrafish, antioxidant, auranofin

## Abstract

Antioxidant supplementation may potentially be beneficial for embryonic development to reduce complications associated with increased levels of oxidative stress. Chlorogenic acid, one of the key polyphenolic antioxidants in *S. oleraceus*, was evaluated for potential protective effects during embryonic development of zebrafish exposed to the teratogen auranofin. Zebrafish embryos were transiently exposed to auranofin to induce developmental abnormalities. Phenotypic abnormalities were scored based on their severity at day 5 post-fertilization. The embryos supplemented with 250 µM chlorogenic acid showed a significantly lower score in phenotypic abnormalities compared to non-supplemented embryos after auranofin exposure. Therefore, supplementation with a low dose of chlorogenic acid showed a protective effect from auranofin-induced deformities and encouraged normal growth in zebrafish embryos. This study provides further support for the potential of using antioxidant supplementation during embryonic development for protection against malformation.

## 1. Introduction

The herb *Sonchus oleraceus* L. (Asteraceae), common sowthistle, has a worldwide distribution and is a rich source of polyphenolic antioxidants, such as chlorogenic acid, chicoric acid, and caftaric acid [1]. When compared to blueberries, a fruit known for its high antioxidant content, *S. oleraceus* showed a higher antioxidant content [2]. Polyphenolic antioxidants have shown in vitro antioxidant activity and an ability to scavenge reactive oxygen species, such as hydrogen peroxide and the hydroxyl radical that causes oxidative stress and damage to cells. There are numerous benefits of dietary polyphenolic antioxidants reported in the literature, including anti-inflammatory, anti-cancer, and neuroprotective activities [3]. In addition, we have shown that *S. oleraceus* leaf extracts were capable of protecting cells from ageing induced by chronic oxidative stress [4].

During human pregnancy, oxidative stress is elevated in the maternal body but is also countered at the same time by the increased production of endogenous antioxidants, such as superoxide dismutase (SOD) [5]. Pre-eclampsia, fetal growth restriction, and miscarriages are associated with reduced SOD levels and increased oxidative stress [5]. These observations suggest that increased oxidative stress has a negative impact on fetal development. Therefore, it was hypothesized that endogenous antioxidants help the body to cope with the increased oxidative stress during pregnancy and antioxidant supplementation may aid normal fetal growth and development.

Zebrafish (*Danio rerio*) have been highlighted as a suitable vertebrate model in which to study biological processes associated with human disease. The zebrafish model is also ideal for studies to investigate antioxidants because they show a demonstrable oxidative response to applied stressors [6]. Newman et al. (2015) applied auranofin to zebrafish embryos to induce oxidative stress by compromising the cellular antioxidant defense. Exposing zebrafish embryos to 5 µM auranofin resulted in teratogenic effects and caused deformities such as reduced skin pigmentation, cerebral haemorrhaging, and jaw malformation [6]. These abnormalities were postulated to occur due to oxidative damage to the embryo’s neural crest, a population of cells that contribute to the development of the craniofacial cartilage and bone, the enteric nervous system, heart, and skin pigmentation [6].

As chlorogenic acid is one of the key polyphenolic antioxidants in *S. oleraceus*, it was evaluated for any potential protective effects during embryonic development using zebrafish as a model exposed to the teratogen and oxidative stressor auranofin. A simple morphological assessment was used to evaluate phenotypic effects. It was hypothesized that developmental defects in the embryo caused by oxidative stress could be reduced by supplementation of the antioxidant chlorogenic acid, thus protecting the embryos from phenotypic abnormalities.

## 2. Materials and Methods

### 2.1. Reagents

Chlorogenic acid (98% purity, predominantly trans from coffee seeds) was purchased from Acros Organics (Geel, Belgium), while auranofin (AFN) (≥98% purity), and dimethyl-sulfoxide (DMSO) was supplied by Sigma (St. Louis, MO, USA).

### 2.2. Zebrafish and Animal Husbandry

Mature zebrafish were obtained from and maintained under standard conditions at the Otago Zebrafish Facility, Department of Pathology, Dunedin School of Medicine, University of Otago. All zebrafish research was approved by the University of Otago Animal Ethics Committee (AEC#48-11, 13/6/11). Transgenic zebrafish Tg (sox10:GFP) with fluorescent green neural crest cells were used to visualize craniofacial structures [7]. The zebrafish were housed in 3.5 L transparent polycarbonate tanks, which were attached to a housing facility with a Central Live Support System (Tecniplast^®^ Aquatic Solutions). They were given daily two dry feeds and one live feed of brine shrimp (*Artemia salina*).

A separation barrier between a pair of male and female zebrafish was removed in the morning to allow for spawning to occur. The resulting eggs were collected with a sieve, transferred into a petri dish (100 mm × 15 mm) containing E3 embryo medium (pH 7.2), and incubated at 28 °C [8]. Only viable eggs were harvested into a single petri dish (100 mm × 15 mm) and used for subsequent experiments.

### 2.3. Toxicity of Chlorogenic Acid

The toxicity of chlorogenic acid was evaluated by supplementing the zebrafish embryos with varying concentrations of chlorogenic acid (0 to 700 µM) in the E3 embryo medium at 6 hpf (hours post-fertilization). Mortalities were confirmed by the lack of any discernible heartbeat in the embryo or larva [6]. The dose was deemed to be toxic if the mortality rate was significantly higher than that of the control group without chlorogenic acid supplementation after 5 days post-fertilization (dpf).

### 2.4. Exposure to Auranofin and Chlorogenic Acid Supplementation

Each zebrafish embryo was an experimental unit. The harvested embryos were evenly divided into petri dishes (100 mm × 15 mm) with no specific inclusion criteria. The embryos received E3 embryo medium (pH 7.2) with either 5 µM auranofin (positive control) or 0.05% (*v*/*v*) DMSO (negative control) at 6 hpf to 120 hpf. The treatments groups received 5 µM auranofin from 6 to 30 hpf that was then replaced with E3 embryo medium with (Transient AFN + rescue) or without (transient AFN) 250 µM chlorogenic acid supplementation until 120 hpf (Table 1). Each treatment was repeated in triplicate. The embryos were then kept at 28 °C until 120 hpf, equivalent to 5 dpf.

### 2.5. Phenotyping and Scores

The zebrafish larvae were visually evaluated at 5 dpf using a fluorescence microscope (Fluorescence Stereo Microscope M205 FA, equipped with DFC490 camera, Leica, Mannheim, Germany). This study was non-blinded. Mortalities were noted as described above and surviving larvae were scored according to phenotypic abnormalities. Phenotypic scoring was based on the presence of abnormal pigmentation, presence of swelling of the region around the heart for pericardial oedema, presence of a dense red region in the head for cerebral haemorrhage, tail kinking for tail malformation, and angle deviation of the lower jaw compared to the larvae in negative control for jaw malformation [6]. Each larva was scored individually from (0) healthy, (1) mild, (2) moderate, and (3) severe malformations in every phenotype. Deceased larvae were excluded from the scoring system.

### 2.6. Statistics

All statistical comparisons were performed with Minitab^®^ (Version 17, Minitab Pty Ltd, Sydney, Australia) using General Linear Model analysis of variance (ANOVA). Post-hoc comparisons were assessed using the Tukey test and *p* < 0.05 was considered to be significantly different.

## 3. Results

### 3.1. Toxicity of Chlorogenic Acid

**Figure 1:**
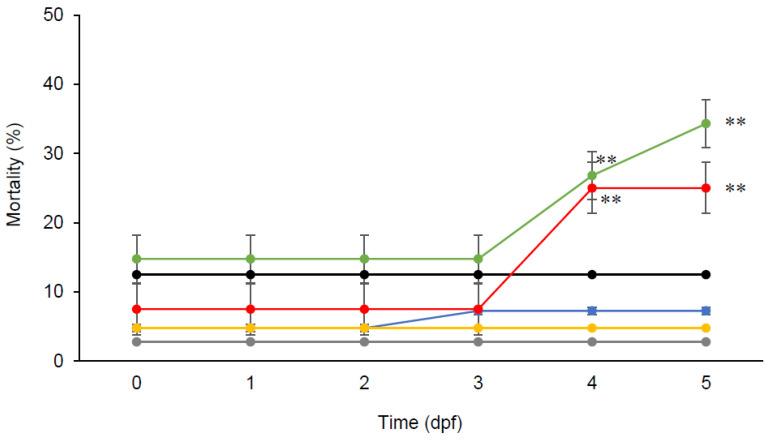
Toxicity of chlorogenic acid (0 µM (•), 50 µM (•), 100 µM (•), 250 µM (•), 500 µM (•), and 700 µM (•)) in zebrafish embryos over a 5-day period. Data presented are means ± SD (*n* = 4, 10 embryos each replicate). ** *p* < 0.01.

Zebrafish embryos were able to tolerate a concentration of chlorogenic acid up to 250 µM for five days, with higher mortalities observed at higher concentrations (Figure 1). Further post-hoc Tukey comparisons in ANOVA analysis showed that embryos supplemented with high doses of chlorogenic acid (500 µM and 700 µM) had significantly higher mortalities (*p* < 0.01) than those supplemented with low doses of chlorogenic acid (250 µM, 100 µM, and 50 µM) and without supplementation.

### 3.2. Effects of Chlorogenic Acid Supplementation

**Figure 2:**
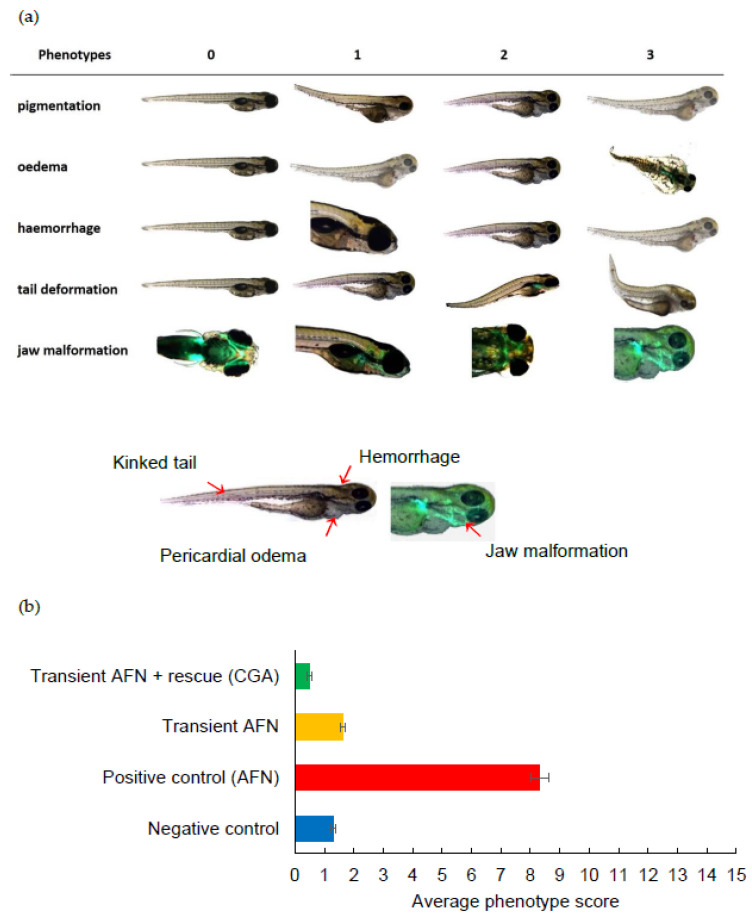
(**a**) Visual scoring chart for phenotypic abnormalities in zebrafish larvae. Phenotype score 0 = healthy, 1 = mild, 2 = moderate, and 3 = severe malformations. (**b**) Average score for phenotypic abnormalities observed in zebrafish embryos at 5 dpf transiently exposed to AFN followed by chlorogenic acid supplementation. Data are means ± SEM (*n* = 84–237). AFN = auranofin and CGA = chlorogenic acid. Groups that do not share the same letter are significantly different (*p* < 0.05). For statistical analysis, degrees of freedom = 3. Reduced pigmentation (F-value = 586.19; *p*-value 0.000), oedema (F-value 294.86; *p*-value 0.000), hemorrhage (F-value = 37.28; *p*-value 0.000), tail malformation (F-value 44.00; *p*-value 0.000), jaw malformation (F-value 199.56; *p*-value 0.000), total malformations (F-value = 393.38; *p*-value 0.000).

After a five-day period, the larvae were evaluated using a visual phenotype scoring system (Figure 2a). The larvae from embryos with continual AFN treatment (positive control group) showed significant abnormalities (*p* < 0.05), including reduced pigmentation, oedema formation, abnormal tail formation, and jaw formation, when compared to the negative control group. Following transient exposure to AFN (6 to 30 hpf) without chlorogenic acid supplementation, a significant reduction in skin pigmentation (*p* < 0.001) and increased jaw malformation (*p* < 0.001) was observed in resulting larvae when compared to untreated controls. With supplementation of 250 µM of chlorogenic acid, transient exposure to AFN led to skin pigmentation and jaw development that was equivalent to controls that were not treated with AFN (*p* < 0.05) and no jaw malformations (*p* < 0.05). The larvae with chlorogenic acid supplementation also showed significantly less severe haemorrhaging (*p* < 0.05) and tail malformation (*p* < 0.05) when compared to other groups.

**Figure 3:**
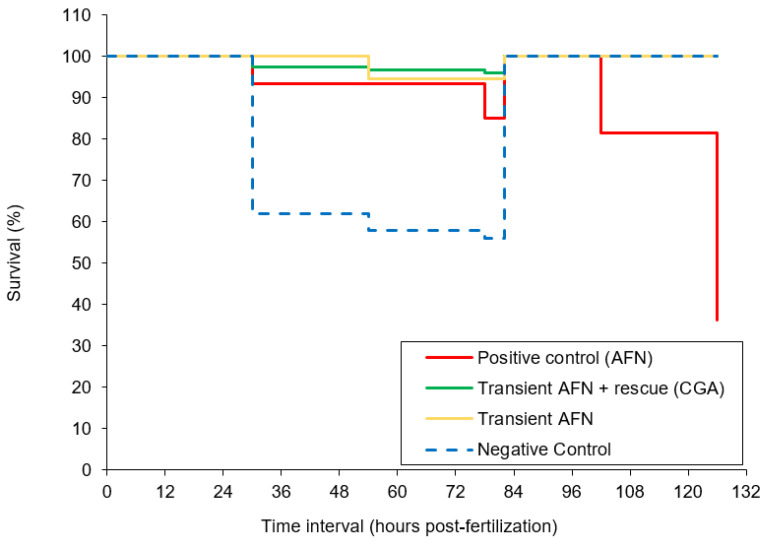
Kaplan-Meier analysis on the survival rate of zebrafish embryos transiently exposed to AFN followed by chlorogenic acid supplementation. Data are means ± SEM (*n* = 84–237). Dechorination occurred at 84 hpf.

All the phenotypic abnormality scores were summed, with a possible maximum cumulative score of 15 indicating a severely deformed larva. The larvae exposed to AFN continuously (positive control) showed the highest deformity (Figure 2b). However, the larvae exposed transiently to AFN had similar average score in deformity to the negative control group (*p* > 0.05). The larvae supplemented with chlorogenic acid after transient AFN exposure showed a significant (*p* < 0.01) reduction in deformity when compared to the larvae in the negative control group and the larvae that were transiently exposed to AFN but did not receive supplementation. It should be noted that the larvae that were transiently exposed to AFN did not show a high mortality rate as opposed to those that had continual exposure to AFN (Figure 3). Continual exposure of zebrafish embryos to AFN (5 µM) resulted in the highest mortality rate of 37% (Figure 3). The negative control with DMSO treatment had a high mortality rate of 56% after 78 hpf (prior to dechorination). Transient exposure (6 to 30 hpf) to AFN had the lowest mortality of zebrafish embryos compared to exposure to DMSO (Figure 3). With 250 µM chlorogenic acid supplementation, there was no increase in mortality.

## 4. Discussion

The increase in oxidative stress, due to transient AFN exposure, has been hypothesized to damage macromolecules due to increased generation of either free radicals or highly reactive aldehydes [9], which may cause malformations in embryos. An increase in oxidative stress, here due to transient exposure to AFN (6 to 30 hpf), caused a significant reduction in skin pigmentation and increased jaw malformations in zebrafish embryos without increasing the mortality rate. Although it was reported that continual exposure to AFN would result in severe phenotypic abnormalities and deformities [6], only significant reduction in skin pigmentation and mild jaw malformation was observed with transient exposure to AFN in our study. Our transient exposure model was more suitable to study the benefits of chlorogenic acid supplementation as the transient exposure to AFN did not affect the mortality rate, while being able to induce deformities.

In the negative control group (DMSO) that was not exposed to AFN, slight mild haemorrhaging and tail malformation were observed. These observations were expected as sporadic embryonic deformities can occur in nature [10]. Highly variable mortality rates have also been reported in embryos exposed to cryoprotectants, such as DMSO [11]. After transient AFN exposure, zebrafish embryos supplemented with 250 µM chlorogenic acid showed minimal phenotypic abnormalities, which was comparable to the negative control group. Further, zebrafish embryos supplemented with 250 µM chlorogenic acid had a reduction in the severity of cerebral haemorrhaging and tail malformation compared to the negative control group. As the total phenotype score was significantly reduced in embryos supplemented with chlorogenic acid compared to the negative control group and the group transiently exposed to AFN without supplementation, it can be concluded that the chlorogenic acid supplementation was able to prevent the malformation induced by transient exposure to AFN and encourage normal embryo development. Therefore, consistent with the literature [5,12], supplementation with antioxidants during embryonic development could potentially provide a protective effect for the embryo against malformations.

Reimers et al. (2006) have cautioned the extrapolation of data on embryonic protection to all antioxidants. In an ethanol-induced stress model, it was demonstrated that antioxidant glutathione and lipoic acid significantly reduced the incidence of pericardial oedema in zebrafish embryos, whereas ascorbic acid and trolox (water-soluble vitamin E) did not [9]. Although the authors postulated that the lack of embryonic protection from certain antioxidants could be due to the low absorption of the antioxidants [9], it is important to note that the antioxidant activity can vary significantly between compounds [1]. It was also brought to our attention that high dose chlorogenic acid (500 µM or greater) was in fact detrimental to the development of the zebrafish embryos to the point of being lethal. Therefore, the appropriate dose of antioxidants should be considered in order to maximize the potential benefits in supplementation during embryonic development.

Antioxidant supplementation during embryonic development has potential benefits. Polyphenol antioxidants extracted from the brown alga *Ecklonia cava* are able to reduce oxidative stress and cell death in zebrafish embryos stressed by ethanol [13]. Similarly, benefits of antioxidant supplementation have also been reported in rats, with the antioxidants vitamin C and butylated hydroxytoluene being shown to significantly reduce congenital malformations [14]. These observations suggest that antioxidant supplementation to the mother can aid in the healthy development of the embryo and that direct administration of the antioxidant to the embryo is not required.

Although oral supplementation of antioxidants in pregnancy may promise benefits to both the mother and the embryo, its effectiveness may be limited by the absorption of the antioxidants. Antioxidants similar to chlorogenic acid, such as silymarin and quercetin, are prone to hydrolysis in the intestine and during first pass metabolism that results in low oral bioavailability of 1% to 4% [15,16]. Therefore, oral formulations, such as nanoparticles or liposomes [17], are required to enhance the oral bioavailability to ensure therapeutic levels of the antioxidants in the body are achieved for clinical benefits.

## 5. Conclusions

We have shown the protective effects of chlorogenic acid antioxidant supplementation in zebrafish embryos exposed to the teratogen auranofin using a simple phenotypic assay. Therefore, low dose chlorogenic acid antioxidant supplementation may potentially be beneficial for embryonic development during pregnancy. As chlorogenic acid is only one of the major key antioxidants in *S. oleraceus*, whole leaf extracts from *S. oleraceus* have the potential to provide further enhanced antioxidant effects. It is important to note that although the benefits of chlorogenic acid antioxidant supplementation were observed in a zebrafish model, mammalian fetal development is different and the benefits of supplementation remain to be further verified in mammals. Further, in order to harness any potential protective effect, oral supplementation of antioxidants would require formulation to enhance oral absorption and bioavailability in humans to achieve the therapeutic level.

## Figures and Tables

**Table 1:** Treatments received by zebrafish embryos.

Group (*n* = 3)	Treatment	Exposure Period
Negative control	10 µM DMSO	6 hpf to 120 hpf
Positive control	5 µM AFN	6 hpf to 120 hpf
Transient AFN	5 µM AFN, followed by E3 medium (blank)	6 hpf to 30 hpf 30 hpf to 120 hpf
Transient AFN + rescue	5 µM AFN, followed by 250 µM chlorogenic acid	6 hpf to 30 hpf 30 hpf to 120 hpf
